# Wisdom from the past

**Published:** 1970-10

**Authors:** 


### Wisdom from the Past

**(Extracts from This Journal 1895 and 1920)**

### SEVENTY-FIVE YEARS AGO

### PROGRESS IN THE MEDICAL SCIENCES — SURGERY

"In support of his theory that the enlargement of the thyroid causes the symptoms of Graves's disease, Wette reports two cases from literature, one described by Professor Hack, and another by Professor Hopmann, in which all the symptoms of exophthalmic goitre were proved to be caused by a diseased state of the nose, disappearing when the trouble in the nose had been remedied. He further points out that the atypical symptoms of Graves's disease, the nervous manifestations such as hysteria, hypochondria, epilepsy, tremor, sleeplessness, are also found in cases in which the exophthalmos is wanting, and that these nervous symptoms are caused by the presence of the goitre. These symptoms are at present ascribed to a toxin produced by the pathological thyroid gland which finds its way into the system. In conclusion, Wette sums up as follows :

1. The principal and surest treatment of Graves's disease is the partial removal of the goitre.

2. The goitre is one of the real causes of Graves's disease :

   A. Of its typical symptoms, exophthalmos and palpitation of the heart, either by pressure on the sympathetic or in consequence of the reflex neurosis of this nerve produced by its terminal branches in the thyroid gland.

   B. Of the complex of aytpical nervous symptoms, the consequence of a general intoxication of the body by chemical metabolism in the pathological thyroid gland.

"Professor Mikulicz, of Breslau, says that operative interference in Graves's disease is gaining ground every day, and no one at present doubts the efficacy of this treatment. He has operated on eleven cases; in all of these tachycardia existed in a very marked degree, and five patients presented serious dyspnoea. All survived the operation, which twice consisted in ligature of the thyroid arteries, while in the other nine cases extirpation was practised. Of the patients operated on, six were completely cured, and in four there was considerable improvement; in one case the improvement was but slight, but in this instance the operation consisted merely in ligature of the thyroid arteries of one side. In the case of one patient, treated by unilateral resection, there was at first great improvement in the morbid symptoms, but the result proved to be ephemeral, for in a short time the tumour on the opposite side developed to such an extent as to compress the trachea. Five months later this side was also extirpated with satisfactory results. The effects of the operation appear much the same, no matter whether the operation be ligature, enucleation, or resection. Operation is by no means free from danger. Although no death occurred in any of his cases, yet he twice met with alarming symptoms, especially rapid pulse and a dyspnoea so intense that he expected to have recourse to tracheotcmy. The effects of operation are manifested within a period varying between a few weeks and several months; but whether improvement takes place at an early or remote period, the patients are invariably satisfied with the results, and justly so. It takes a long time for the cardiac disturbances to subside and the exophthalmos to disappear, but this is not surprising. The favourable effect of operation on Graves's disease has been explained in various ways. It cannot be attributed to relief of pressure on the trachea, for dyspnoea is often absent. Nor is the theory of the cervical sympathetic being injuriously influenced by the tumour tenable. It is more likely that there is auto-intoxication by the products of secretion of the hypertrophied thyroid gland, while it is more probable still that the disease begins as a neurosis, and that the symptoms are gradually aggravated by exaggerated secretion of this organ. Professor Kocher, of Berne, considers that the operation of extirpation of goitre in Graves's disease is dangerous, for he has lost three cases; and he has since had recourse to ligature of the thyroid arteries, taking care never to tie more than three of these vessels, because in one case in which he ligated all four arteries the operation was followed by symptoms of cachexia. Professor Krönlein, of Zurich, prefers extirpation of the gland, which he has performed successfully in eight cases. The exophthalmos persisted in one case, but the patient was, nevertheless, well satisfied with the result of the operation. Professor Trendelenburg, of Bonn, has, in some cases of Graves's disease, had recourse to ligature of the thyroid arteries, invariably tying all four, in two sittings, at an interval of a few weeks. He has never met with symptoms of cachexia strumipriva. Professor

Rydygier, of Cracow, has practised ligature in twenty-two cases of Graves's disease. He has tied all four arteries without ever noticing any symptoms of cachexia.

— **Vol. 13, pp. 282-283 (1895)**

### FIFTY YEARS AGO

"One of the most urgent and crying needs of the day in connection with the Bristol voluntary hospitals is the institution of a properly-equipped Almoner Department, common to all the hospitals. It would prevent the philanthropic subscriber feeling that perchance his self-denial is misplaced and that his contributions are filched by patients who can quite well afford to provide for their own needs.

"An Almoner requires special training in work which is highly responsible, and calls for tact, knowledge of the work, and organising capacity, and when, as in Bristol, there are several hospitals, it is essential that the Almoner's Department of each should be working in collaboration under one head, if reduplication and waste of money and effort is to be avoided. Furthermore, the department should ensure the equal collaboration of the lay committees and the honorary medical staffs of the various institutions. The work of such a department is by no means confined to sifting out patients whose financial status renders them improper persons for treatment in a voluntary hospital, for the inquiry reveals and provides for many cases where there is a call for sympathy and help outside mere treatment. The heads of the department would be cognisant of all the manifold charitable organisations in and around the city, and would constitute an invaluable source of information to many a poor patient, and to many who want to get into touch with those who really need help."

— **Editorial Notes. Vol. 37, pp. 232 (1920)**

